# Assessment of Noise Exposure in United States Urban Public Parks: A Scoping Review

**DOI:** 10.3390/ijerph22121882

**Published:** 2025-12-18

**Authors:** Ugoji Nwanaji-Enwerem, Kevin M. Mwenda, Shira Dunsiger, Diana Grigsby-Toussaint

**Affiliations:** 1 Department of Behavioral and Social Sciences, Brown University School of Public Health, Providence, RI 02903, USA; shira_dunsiger@brown.edu; 2 Warren Alpert Medical School of Brown University, Providence, RI 02903, USA; 3 Institute at Brown for Environment and Society, Providence, RI 02912, USA; kevin_mwenda@brown.edu; 4 Population Studies and Training Center, Brown University, Providence, RI 02912, USA; 5 Spatial Structures in the Social Sciences, Brown University, Providence, RI 02912, USA; 6 Data Science Institute, Brown University, Providence, RI 02912, USA; 7 Department of Biostatistics, Brown University, Providence, RI 02903, USA; 8 Center for Health Promotion and Health Equity, Brown University School of Public Health, Providence, RI 02903, USA; 9 Department of Epidemiology, Brown University School of Public Health, Providence, RI 02903, USA

**Keywords:** parks, noise, sound, recreation, public health

## Abstract

Adverse exposure to noise pollution is increasingly recognized as a significant public health concern. Strong evidence links noise exposure with negative health outcomes such as cardiovascular disease, mental disorders, stress, and sleep disturbance. The presence of noise in parks, which are environmental settings associated with health promotion, recreation, and restoration, presents a paradox that warrants further exploration. The United States offers a distinct context for exploring this paradox, given its vast public park system and a wide array of anthropogenic and environmental noise sources. Our scoping review synthesized findings from fifteen research studies that investigated noise exposure and noise levels in United States public parks. The review examined how studies measured noise, the integration of subjective perceptions with objective assessments, and the role of park characteristics in shaping park visitor noise experiences. Results highlighted varying methodological approaches, with some studies employing sound level meters or modeling techniques, while others also incorporated surveys to capture visitor perceptions. Despite this variety, evidence on the direct health impacts of park noise exposure remains limited, and longitudinal studies are largely absent. Notably, few studies evaluated how noise interacts with other environmental exposures, such as air pollution or greenness, to influence visitor perception and wellness. By synthesizing the current evidence base, this review suggests knowledge gaps and few methodological inconsistencies that limit the field. Findings call for future research mobilizing standardized, multimodal noise assessment methods, and considerations for health outcome measures. Such advancements are important for informing public health interventions and guiding urban planning strategies to improve the acoustic quality and restorative potential of US parks.

## 1. Introduction

### 1.1. Overview of Environmental Noise Exposure and Effects on Health

Noise is defined as unwanted sound that can harm the health, safety, and well-being of living organisms, including human populations [1]. With increasing urbanization of society, noise pollution is classified as the second most significant environmental threat to health, resulting in a range of adverse auditory and non-auditory health effects [2]. In the United States (US), an estimated 1 in 2 individuals (50% of the population) are exposed to harmful noise exposure according to Hammer et al. (2014) [3]. This study and Seidman and Standring (2010) report that over 145.5 million people are cited to be at potential risk of hypertension due to noise exposure, and an estimated 10 million adults and 5.2 million children are diagnosed with irreversible noise induced hearing impairment, respectively [3,4].

The subjectivity of noise perception is relevant to public health, as research suggests that physiologically it is performed through the perception of noise and noise annoyance that elicits the autonomic nervous system, which induces the stress response [5,6,7]. Depending on the duration and characteristics (level, loudness, peak exposure), noise-induced stress can trigger adverse health effects from cardiovascular risk to metabolic dysfunction [8,9]. Though a primary focus of hazardous noise exposure is common in occupational settings, noise exposure permeates in several other environmental contexts, including residential areas and social spaces [10]. Within urban settings, exposure to noise also occurs in places typically characterized as quiet havens, such as parks [10].

### 1.2. Parks and the Presence of Noise Exposure

Parks are built environment settings characterized as spaces containing natural vegetation that are open and accessible to the public [11]. Parks, which are considered a form of greenspace exposure, commonly function as environmental areas for recreation and relaxation, facilitating health promotion in the population [11]. Parks serve as a platform for the adoption of healthy behaviors, including exercise, meditation, and socialization [12]. However, the capacity of parks to serve as a setting for health promotion depends on how conducive the features, design, and functionality of the park are. Thus, the quality of parks strongly influences their capacity to be utilized for healthy behaviors (i.e., physical activity) [13]. Park quality is often contingent on perceived factors and characteristics that can be sensed (i.e., visual observations, auditory conditions) such as park landscape maintenance and park soundscape characteristics which in totality impacts the park visitors’ subjective experience in the space [13,14,15]. Further, the presence or absence of certain factors can heavily impact park users’ health while in the park environment.

Among these perceived factors, sound exposure is an invisible yet ubiquitous facet of the environment [16,17]. Sound is scientifically characterized as a pressure wave that travels in time and space and carries energy and information [16,17]. The capacity of sound to “carry energy and information” is important for defining noise and characterizing its potential danger in settings such as parks. While not all sound is inherently harmful, certain sounds when perceived as intrusive can trigger annoyance and stress, both known to have adverse health effects. This makes their presence in parks, spaces visited for health promotion, a contradictory phenomenon.

### 1.3. Sound: The Good and the Harmful

Leaves rustling and birds chirping are often described as “nature sounds” that foster the sentiment of serenity and relaxation afforded by green environments [18]. Sounds confer perceptiveness of the environment, connecting humans with nature [19]. Health benefits have been found to be associated with the soundscape of nature, which offers evidence for the restorative capacity of acoustic environments within parks [19,20]. However, the soundscape of parks consists of the summation of all perceived sounds including those from biological sources (i.e., animal sounds), geophysical sources (i.e., wind and rain), and anthropogenic sources (i.e., road and air traffic noise). Sounds from anthropogenic sources are often perceived as noise (i.e., “unwanted sound”) that therefore disrupt natural sounds. The dominance of anthropogenic sound can mask natural sounds and result in an altered perception of the soundscape that can be negative to health [14,18].

The growing prevalence of anthropogenic noise in urban environments has heightened concern about its impacts on specific sub-components of cities, such as urban parks. National parks are likewise affected, with one-third located in urban areas (National Park Foundation, 2025). In parks, which are commonly visited to relieve stress and relax in a tranquil atmosphere, it is important to identify research measuring the presence of the environmental stressor of noise and its downstream effects [21].

To date, there has been minimal research that synthesizes evidence on the presence and evaluation of noise in parks in the US. A 2023 review conducted by Rey-Gozalo et al. analyzed the role of greenspace in mitigating adverse noise, with a focus primarily on studies conducted in European cities [22]. In addition, numerous studies have been conducted in other countries including those in South America (e.g., Brazil), where the primary motivation for the evaluation of noise levels in parks was to assess compliance with environmental protection legislation and noise level regulations [23]. To our knowledge, no study has synthesized the body of literature that investigates environmental noise in urban park environments in the United States. In the United States, the regulation of noise and cultural acceptability and annoyance perception of noise varies from other countries in the world [24]. The geographical variation in sound profiles, noise sources, and park infrastructure warrants a place-specific exploration of the literature investigating noise exposure in US parks. Additionally, US park systems differ from other countries in purpose, design, and use patterns. The predominance of recreation and the type of athletics in parks spaces, warranting certain park features (e.g., basketball court), climate and season variations, and popularity of factors such as pet ownership are important considerations related to park activity use in the United States that are distinct. A 2024 study examining thirty-five cities across the globe found that United States parks emphasize physical activity amenities in contrast to European parks that focus more on nature appreciation features [25]. Recognizing these variations highlights the importance of examining studies that evaluate noise exposure in US parks, to derive context-specific information and recommendations.

In US parks, the reality of how park characteristics both objectively affect sound levels and also the human perception of noise is under-explored. The extent to which studies evaluating park sound levels also examine relationships between green space characteristics and noise has not been synthesized, leaving an important gap in understanding. Given the goal to map the existing evidence base and identify knowledge gaps in noise in park assessment studies conducted in the US, a scoping review was selected as the most appropriate methodological approach. This scoping review aims to explore which studies investigate noise in parks objectively, the utilized methodology to measure noise, and also which studies address the subjectivity of noise as unwanted sound and include procedures to examine park user’s opinions about the park quality with regard to sound.

The goals of this scoping review are as follows:

1. Summarize the evidence of studies investigating noise in urban parks in the United States
2. Synthesize and discuss key methodological approaches of studies, including those that evaluate the subjectivity of noise exposure on park visitors’ experience
3. Provide insight on knowledge gaps across studies pertaining to research and public health implications of noise in parks, including insufficient data on effects of park noise on park users or absence of studies assessing stress-related health outcomes

Moreover, this review paper has important implications for environmental health and inferences for summarizing the existing evidence that is instrumental for informing public health law and policy aimed at protecting and enforcing compliance of noise regulations in open spaces such as parks.

## 2. Methods

This scoping review is consistent with the PRISMA-ScR (Preferred Reporting Items for Systematic Reviews and Meta-Analyses extension for Scoping Reviews) methodology. A registered protocol was not used for this review. The PRISMA-ScR methodology is a systematic approach for coalescing and reviewing evidence based on key themes and existing information [26].

### 2.1. Eligibility Criteria

Quantitative studies, including cross-sectional studies in parks located in the United States (US), were included in this review. To be included, studies had to explicitly take place in public US parks (i.e., urban parks, national parks in urban areas) and evaluate noise, based on the Environmental Protection Agency (EPA) definition of “unwanted or disturbing sound that interferes with normal activities such as sleeping or disrupts or diminishes quality of life” through primary data collection [27]. Studies had to objectively measure noise and explicitly name the research setting of parks in the title and/or abstract and/or keywords. Studies using secondary data and/or not describing the methodology used to conduct noise measurement were excluded. In addition, studies were required to have a quantitative design component; a control or comparison group was not required. To meet the inclusion criteria, studies had to involve measurement of noise in parks. In addition to noise levels, the secondary outcome variable considered but not required in this review were park visitor perspectives which encompass perception on park visitors acoustic comfort, satisfaction, annoyance, etc.

In summary, studies eligible for inclusion in this review met the following criteria: (1) were quantitative studies that (2) had explicit components on noise exposure measured either solely through objective methods or through a combination of objective and subjective methods (3) and took place in any public urban area park in the United States. For this review, public urban area park is defined according to Zhao et al. (2024) as “large green spaces [within a city or metropolitan area] intended for recreational use that is accessible for all residents and visitors” [28].

### 2.2. Search Strategy

A comprehensive literature search of the following databases was conducted: Scopus, Science Direct, Web of Science, and PubMed. Search strings for noise pollution and environmental noise, urban parks, and urban greenspace were developed utilizing validated search strings from previously published reviews and knowledge of noise pollution and parks. The search strings also included Medical Subject Headings (MeSH), which are used for indexing articles in PubMed. The search strings were combined for sound, noise, and urban parks and national parks. The complete search strategy can be found in Supplementary Materials S1. The finalized search strategy was also confirmed with consultation from a Brown University librarian. Across all databases, 3324 studies were imported into Covidence, which identified and removed 901 duplicates [29]. The finalized search resulted in 2423 studies to be screened.

### 2.3. Study Screening Process

**Figure 1:**
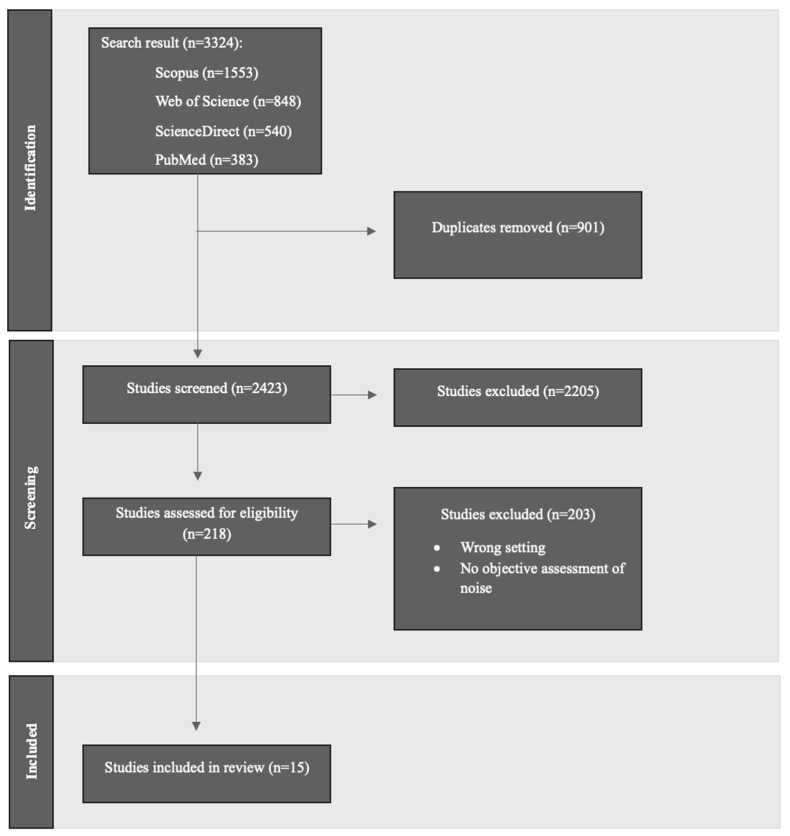
Flow diagram of article selection.

For each study, two authors reviewed the title and abstract. Studies were screened using the following Population, Exposure, Comparison, Outcome, Study Design (PECOS) inclusion criteria: (population/place (parks); exposure (noise); comparison; outcome (noise levels, noise perception); study design (quantitative)). Studies that failed to meet the inclusion criteria were not selected. Out of all screened studies, two authors only had a discrepancy over two articles which was resolved through meeting and having a conversation using the PECOS criteria to finalize the decision of whether to move the article to full text. If both authors were still uncertain about whether a study met the inclusion criteria after reviewing the title and abstract and subsequent discussions, the study was then moved to full-text review. The inclusion criteria were successfully met by 218 studies which were moved to full-text review. In the full-text review, the PECOS inclusion criteria were used to select and exclude studies. Fifteen studies were included in the final synthesis and will be summarized in this review. The full PECOS screening checklist used in the study inclusion process can be found in Supplementary Materials S2. The PRISMA flow diagram displaying the study selection process is shown in Figure 1.

### 2.4. Data Extraction Process

Critical information was extracted from all studies included in the final synthesis. The authors summarized the studies, synthesized the results, and critiqued the studies’ methodological quality. The authors read each of the studies in their entirety and extracted the following information: authors; publication year; study location; park name; study duration; study methods; relevant findings; study considerations; and discussed limitations (Table 1). Data regarding the dimensions of noise measured were also extracted for each study (Table 2). Though the study is a scoping review, to increase the strength of the assessment of the evidence base, methodological critique was conducted for each study using the AXIS Critical Appraisal Tool (Table 3). The authors noted other important information relevant to the topic area such as study limitations in the summary tables.

### 2.5. Critical Appraisal Process

Studies were critically appraised using the Appraisal Tool for cross-sectional studies (AXIS). These tools are useful for assessing internal validity and were established to aid reviewers’ assessment of a study’s risk of bias [30]. The AXIS Appraisal Tool for Cross-sectional Studies is a 20-question checklist, with answer choices of yes, no, or do not know/comment. The authors will complete each question for the cross-sectional studies included in the final synthesis and make a global rating of either weak, moderate, or strong.

### 2.6. Synthesis Process

For synthesis, the authors extracted critical information from each of the included studies and the critical appraisal. Consideration of the number of qualitative, quantitative, and subjective dimensions of noise assessed in each study were made. These dimensions included the following: Noise level, usually measured in decibels, are logarithmic units that can be used to compare wide ranges in sound intensities [31]. Noise event is the sequence of an audio clip received from an action, such as sound patterns, people speaking for a few seconds, etc. [32]. Noise type and noise source refers to what person, place, or thing creates, is the origin, or emits noise, with categorization as either from nature (i.e., geophonic), from a non-human living organism (i.e., biophonic), or from humans (i.e., anthropogenic) [33,34].

Noise sensitivity is the individual variation in the effects of noise that may be viewed as both and inter- and intra-personal effects [35]. Perception of noise and attitudes toward noise exposure refers to the subjective experience of noise which depends on the characteristics of the person, place, activity, and their interactions in space and time [34]. Noise audibility is the capacity of sound to be perceived by an animal with normal hearing and is influenced by the hearing ability of the animal, the masking effects of other sound sources, and by the frequency content and amplitude of the sound [36]. Noise annoyance is a stress reaction to environmental noise and refers to a subjective parameter considered to reflect the internal exposure to noise [37].

The authors then synthesized the study exposure approaches and key implications of findings. The results from this synthesis are reported with three underlying themes from the studies.

## 3. Results

The search identified 3324 articles across Scopus, Web of Science, ScienceDirect, and PubMed. Following screening and full text review, fifteen studies were included in this review (Figure 1, Table 1).

### 3.1. General Study Characteristics

The studies included in this review were published between 2004 and 2024. All studies were conducted in the US with the majority (53%) of studies taking place in parks in New York [38,39,40,41] or California [14,42,43,44]. One of the studies included in this review occurred in Texas, which was the only southern state of the United States represented among included articles [45]. One of the studies occurred in the New England region of the US, investigating noise in parks in Boston, Massachusetts [46]. In addition to the studies that were conducted in New York, the Mid-Atlantic was also represented in this review through a study conducted in Pennsylvania parks [47]. Variability also existed across studies based on park characterization, with all studies in this review taking place in parks categorized as public parks; however, 53% of the studies took place in parks specifically classified as national parks. While the broader aims of all the papers included in the review sought to investigate noise in the context of a park environment, there are important distinctions behind the motivations for each study that extended in purpose beyond solely measuring noise in parks. Taff et al. (2014) investigated whether educational messages informing park visitors about the presence of military aircraft influenced their acceptability of the resulting noise in the park environment [44]. Carter (2014) and King et al. (2018) sought to generate maps, the latter specifically aiming to develop a map of tranquility, with both studies coupling noise level data and photographic images of parks [39,41]. Lynch et al. (2011) and Rice et al. (2022) had study aims that were primarily focused on generating insights to inform park management decisions on how to better manage noise exposure within the parks [36,43]. The exposure variable for all studies was noise; however, source and types of noise were also distinctions amongst papers, with three studies specifically focusing the primary research objective on assessment of the impact of aircraft noise in the park environment [44,47,48]. Four studies in the review involved a park visitor survey component, all of which took place in California parks [14,42,43,44].

Though the time of year was not explicitly mentioned for all studies, seven of the studies (47%) conducted assessment of noise in parks in the summer months [14,42,44,45,46,48,49]. Three of the studies (20%) conducted assessment of noise in the autumn season [40,43,46]. Only one study explicitly mentioned the use of data across all months of the year [50].

Study duration varied across papers, with the shortest study period reported being three days long [38,40]. Multiple studies noted the specific presence of anthropogenic sources of noise, including noise from road traffic and aircraft [14,36,42,48,50]. Some studies focused on the value provided by parks in protecting pedestrians in cities from increased exposure to noise, as alternative routes away from noisy roads [40,45]. Other studies emphasized the implementation of interventions, such as quiet pavement or rerouting flights above parks to mitigate the presence of noise in parks [43,47].

**Table 1:** Overview of included studies.

Author (Publication Year)	Study Location/ Funding Source If Specified	Park Name/Park Type	Study Aims	Study Duration and Methods	Key Findings and Conclusions
Bourdeau et al. (2015) [38]	New York City, USA University of Hartford under the Greenberg Junior Faculty Grant 2014–2015.	The High Line/Urban Public Park	To assess the acoustic characteristics and pedestrian exposure to noise on the High Line.	Three days Fixed spot and in-transit noise measurements using sound level meter.	The study captured the average noise exposure of pedestrians walking in the park with support that greater distance of pedestrians from road traffic can reduce pedestrian exposure to noise pollution by up to 4.6 dB. Authors comment on the utility of future work that assesses the attitudes to the High Line soundscape that can further quantify how to enhance the quality of the park. Active construction sites observed and likely to have impacted the noise levels.
Buxton et al. (2019) [50]	USA	US National Parks	To diagnose noise levels and sources across park units and summarize results from continental-scale models.	Study duration not clearly specified, though supporting tables report data collected across winter, summer, fall, and spring seasons. Spectrograms of noise recordings from 251 sites in 66 park units were obtained, and for 168 sites, categories of noise were characterized. The audibility among noise categories fit into a generalized linear model. Noise exceedance and comparison across park types were explored.	Parks with increased road density and close in distance to airports experiencing higher number of noise events. Aircraft and road vehicles were the most common sources of noise, but trains and watercraft, when present, created the loudest noise levels in parks.
Lynch et al. (2011) [36]	USA	US National Parks	To present monitoring and analysis protocols, summarize the acoustic conditions, and identify key patterns. To discuss ways parks have incorporated noise data into management actions.	Twenty-five days Noise data were collected at 189 sites in 43 national parks. Offsite listening and visual analysis to identify sound sources was conducted primarily. Audibility analysis was performed to determine how often anthropogenic sounds were perceptible by humans at each site, involving the manual logging of noise events and calculations of natural ambient sound level.	The quietest sites in the dataset have audible noise greater than 5% of most daytime hours. Most sites had high noise audibility from 0700 to 2200 h. The pattern of noise audibility in the sampled parks reflects the activity cycles of humans was found to be nearly identical to pattern of aircraft noise. The quietest sites in parks are the most vulnerable to noise intrusions.
Betchkal et al. (2023) [48]	Hawaii and Alaska, USA Natural Sounds and Night Sky Division of the US National Park Service (Task Agreement P21AC10586)	Hawaii Volcanoes National Park (HAVO) and Denali National Park (DENA)	To pair aircraft tracks with acoustic data to understand the effect of aircraft noise sources and to determine at what distances the functional effects of aircraft noise might begin to affect park environments.	For HAVO, five days of intersection data from June to September 2019 and for DENA, forty-nine days of data between May and August 2019. Quantitative observation-based audibility modeling was used to pair simultaneously collected aircraft tracking data with acoustic data and estimate the geographic scope of noise impacts for low-level overflights above parks. Acoustic data were collected using a sound level meter. For HAVO, 187 aircraft noise events were obtained and for DENA, 250 noise events.	For each park there was evidence of geographic persistence of aircraft noise audibility. For HAVO, low-level flights within one mile of a receiver could be loud enough to disrupt speech at 5 m. For DENA, aircraft within approximately 1.5 miles of a receiver could be loud enough to disrupt speech at 5 m. The audibility of noise in HAVO and DENA could help to better understand how rerouting flights could change the acoustic environment at a listener’s location within the parks and inform important park management decisions.
Carter (2014) [39]	New York City, O’ahu Hawaii	Two northern Manhattan parks: Ft. Tyron Park and Inwood Hill Park Central community park in O’ahu/Urban Parks	To present a new method of linking the aural and visual conditions of a soundscape to transect maps across complex sonic environments.	Duration not specified. Cross-modal recording method was used linking ambisonics audio capture with sound level meter data with high dynamic range photography to document the soundscapes of the selected urban parks.	In both case studies, the directional audio information attached to the photographic field offered a salient depiction of the specific characteristics of the soundscape; in New York parks, specifically, the roadway sounds are shown to have a large geographical footprint. It is stressed that this multimodal approach of collecting data is key in providing a direct opportunity for researchers to witness the perceptual phenomena in the measured environment that may not be captured by a single measurement device.
Ferguson et al. (2024) [14]	Mill Valley, California, USA National Science Foundation (CNH 1414171)	Muir Woods National Monument/National Park	To understand what factors influence park visitors’ perceptions of park soundscapes.	Surveys were collected between 9 May and 21 May 2016. Nine acoustic recording devices were placed in the park to capture sound levels. To estimate visitor use, automated infrared visitor monitors and manual count calibrations were used. Survey data were collected (*n* = 537) to evaluate visitors’ perceptions of the park soundscape, analyzing pleasantness, noise sensitivity, and noise interference.	Noise interference (impacted by anthropogenic sound sources) more than sound pressure level better explained the perception of the soundscape and noise sensitivity was a significant predictor of soundscape pleasantness. The findings suggest that an individual’s exposure to noise impacts perceptions of a park soundscape which has important implications for park visitors in urban areas and how this affects parks with the goal to provide natural and more restorative soundscapes.
King et al. (2016) [40]	New York City, USA University of Hartford under the Greenberg Junior Faculty Grant 2014–2015	High Line Park/Urban Public Park	To conduct a combined assessment of noise and particulate matter pollution for pedestrians in the High Line park compared to those walking on a footpath alongside road traffic.	Testing was performed over three days (Friday to Sunday) in autumn 2014 (September–October). Noise and air quality measurements were conducted simultaneously by two study participants. Thirty-five pairs of noise and sixty-one pairs of air quality measurements were taken across three separate days. Noise measurements were taken with sound level meters using fixed spot and mobile measurements, with spot measurements being 10 min in duration. Air quality measurements were taken using Aerosol Monitors.	The park was shown to have a positive environmental effect for its users for both air and noise pollution, which suggests value of park environments in protecting city residents and pedestrians from adverse exposures. The local effects of New York City and the dense population and terrain could have affected the particulate matter concentration that may have impacted long-term average concentrations and thus may have led to uncertainty in the reported results. Also, uncertainties are possible in the results due to considerations of street geometry and pollutant hot spots and atmospheric conditions such as wind patterns and local meteorology effects that can impact noise propagation and air pollution which was not accounted for.
King et al. (2018) [41]	New York City, USA	Central Park/Urban Public Park	To develop a map of tranquility within Central Park utilizing measured noise levels and visual features.	Duration not specified. Conducting predefined walks and recording noise levels and using a smartphone app to assess tranquility: noise levels were recorded with dosimeters, locations were logged using GPS enabled smartphones, and photographs were taken at regular intervals with noise events being logged on a smartphone app.	Two optimum tranquility trails were developed: the north path takes walkers away from the city up the Great Hill for skyline views and along the peaceful loch and into the conservatory garden ending at the edge of the Harlem Meer. The south path begins and ends at the Metropolitan Museum of Art going through the Lake and the Mall. The study conducted was not a complete assessment of Central Park and factors including the duration of sound level measurements beyond one-minute intervals. Additionally, the evaluation approach of sound did not account for the context of sound sources within the park.
Levandowski et al. (2021) [49]	Wyoming, USA GRYN, Montana Institute on Ecosystems, University of Wyoming—NPS Research Station, and the American Association of University Women. Scholarship from Veteran Services at Montana State University.	Grand Teton National Park	To explore what additional insights could be gained about wetland biodiversity using cameras and acoustic recorders	Early and late summer 2017 (June–September). Within the park, wetlands were selected and infrared cameras and song meters and acoustic microphones were used.	Utilizing the recordings and software, 8 bat species were identified, which contributed to an important knowledge gap about the understanding of the ecological community in the national park. Capturing the acoustic environment can serve to provide information on the variety of species and document important ecological patterns.
Pilcher et al. (2009) [42]	California, USA Funded by Golden Gate National Recreation area and the NPS Natural Sounds Program Office.	Muir Woods National Monument	To identify soundscape-related indicators by addressing the sounds visitors hear at Muir Woods and the extent to which those sounds are judged to be pleasing or annoying.	July–August 2005 A two-phase study. Park visitors (*n* = 280) were surveyed at three locations along the main park trail. Sound recordings from the park were then used and visitors were asked to listen to each sound clip and rate its acceptability, and the types of sounds heard. Sound recordings of the parks were recorded with shoulder-mounted omnidirectional headphones and fabric windscreen domes.	Rising levels of visitor-caused sound and decreasing levels of natural sounds were increasingly unacceptable. As the decibel level increased, acceptability decreased. Natural sounds were reported to be pleasing, and visitor-caused sounds (e.g., groups talking) were found to be annoying. The results of the study are indicative of a threshold of which park visitors find visitor-caused sound as unacceptable. The findings aid in generating noise-related indicators and standards of sound quality in parks and related areas. The sample only included perceptions of current park visitors who may have incomplete knowledge of the policy contexts of parks and park soundscapes.
Rice et al. (2022) [43]	California, USA Funded by the National Park Service, Natural Sounds, and Night Skies Division (NSNSD) through the Cooperative Ecosystem Studies Unit network.	Death Valley National Park	To examine the efficacy of quiet pavement strategy of noise reduction, assessing how park visitors navigate the tradeoff between quiet and freedom, and to what degree visitors support this park noise management action.	Twenty-one days during the autumn of 2018. A quantitative surveying approach was used that assessed responses of 667 park visitors and conducted a choice experiment involving use of sound data collected by the National Park Service and four attributes representing quietness and freedom, with three levels assigned to each attribute.	Recreationists need significant increases in quietness to relinquish freedom and quiet pavement, and reduced speed limits have the least negative effect on park user/recreationist utility. There is additional need for work assessing and considering the demographics and noise sensitivity of park visitors prior to implementing certain park management decisions.
Sheikh and Uhl (2004) [47]	Pennsylvania, USA	Pennsylvania State Parks	To record the number of aircraft overflights and the audible duration of aircraft noise in state parks in Central Pennsylvania, USA.	Weekdays from 1999 to 2000. Aircraft noise was measured on weekdays for three hours in 18 parks in listening stations and noise was expressed as the precent of time an observer heard aircraft noise during the hour of sampling.	There was an average of 14 overflights per hour in the 18 parks studied. Noise was sampled more from jet than propeller aircraft. Aircraft noise was heard an average of 40% of the sampling period in the 18 state parks. Aircraft noise had a maximum of 41% corresponding to 34 noise events during the sampling period for a park. A relationship was found between noise duration in parks and airport density within set distances from parks.
Taff et al. (2014) [44]	California, USA Funded through the National Park Service.	Sequoia National Park	To inform social indicators and standards and to determine if indirect management actions in the form of educational messaging could significantly affect visitor acceptability of military aircraft sounds.	Summer of 2011. A two-phase study. Phase one involved a visitor survey to determine what visitors were hearing. Phase two involved assessment of sound clips to evaluate visitor standards related to aircraft sounds in Sequoia. A sample of 146 visitors were surveyed. Sound recordings were conducted with a recorder and sound level meter.	Sound recordings with predominately natural sound were found to be very acceptable among visitors but hearing aircraft noise was less acceptable and unacceptable among visitors. Messaging informing visitors of the presence of military aircraft increased visitor acceptability of aircraft sounds by as much as 15%. The study showed that a theoretically based and tested message could be incorporated into a park unit to influence park visitor attitudes, perceptions, expectations, and average thresholds of acceptability for military aircraft sounds.
Terry et al. (2021) [46]	Boston, Massachusetts, USA	Blue Hills Reservation, Hammond Pond Reservation, and Hall’s Pond Sanctuary	To understand noise pollution in parks and protected areas and assess if sound levels were quieter during the pandemic.	Pre-COVID-19 measurements taken in September and October 2017, and July and August 2019; pandemic measurements were taken from March to April 2020 and in late May 2020. Sound level measurements were taken pre- and post-pandemic using the SPLnFFT app with iPhones.	For each park, a change in sound level during the pandemic was observed; however, the direction of change varied across each park based on factors such as roads, the presence of leaves on the trees, and extent of human activity. Road traffic noise was the primary source of elevated sound levels at the study sites. Findings showed that by limiting human activity, the early COVID-19 lockdown in Boston reduced two primary sources of noise.
Yildirim and Ozdil (2016) [45]	Dallas, Texas, USA	Klyde Warren Park (KWP)	To evaluate the park sound levels to assess the distribution and impacts of sounds in urban landscape and to review available soundscape measurement tools.	Summer of 2015 (July–August) Systematic on-site sound recordings on evenly distributed nodes on hypothetical grid overlaid on KWP, with measurements conducted on weekdays and weekends using convenient sampling for fifty-one sound level measurement points.	The sound level measurements of the park ranged from 62.1 to 96.5 dB, with the weekday average being 70.2 dB and the weekend average being slightly higher at 71.2 dB. The variations in sound levels across different parts of the park highlight the importance of sound consideration in park design. The east part of the park was relatively quiet and had amenities such as pocket gardens and small lawns in contrast to the west part of the park.

### 3.2. Synthesis

Based on the full-text review of each article, three themes were developed that summarize key evidence derived from the studies. The first theme involves characterization of the exposure metric, noise, and the diversity in dimensions of noise measured across studies. This theme is vital given emerging research that emphasizes that the complexity of noise requires robust measurement of its various components [51]. The second theme involves research studies in the review that framed parks as settings for experience with a focus on park visitor perceptions and opinions. Extending from the importance of theme one, theme two delves into the evidence obtained from the studies assessing the exposure of noise from a human-centered vantage point. The literature that highlights the peril associated with adverse noise exposure on human health raises the importance of research considering the *perception of noise among humans*, which may be a key predictor of broader health outcomes [51]. The third theme explores the scope of implications offered from the reviewed studies with regard to various disciplines involved in park management and regulation of environmental health exposures in parks.

### 3.3. Theme One: Approach to Noise Exposure Measurement and Variation in Assessed Exposure Characterizations

The literature suggests that predominant measurement approaches to assess noise as an exposure may fail to also investigate other relevant parameters associated with the exposure that may be strong correlates of degradation of the quality of urban environments and predictors of adverse health outcomes [51]. To illustrate the variability in parameters of noise measured, Table 2 was created to portray the dimensions of noise measured in each study included in the review. The dimension of noise is labeled for each column.

**Figure 2:**
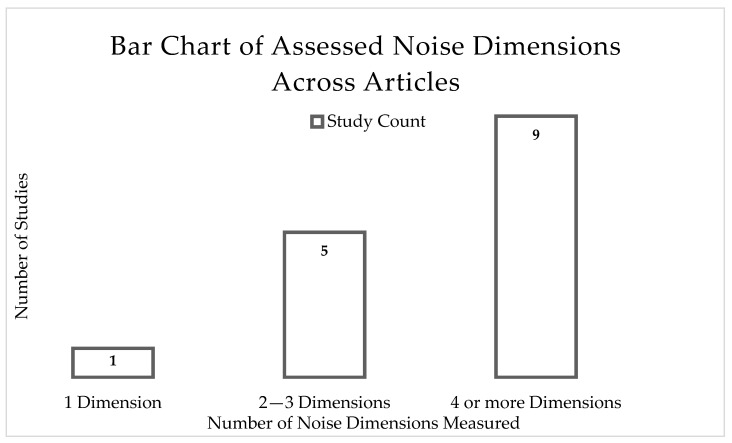
Summary of studies and number of noise dimensions measured.

Across 14 of the 15 included studies, noise levels of each park were reported as the primary dimension of noise exposure measured. The one study that did not report noise levels in the study expressed noise exposure through audibility, quantifying the exposure as the percent of time an observer heard aircraft noise during the hour of sampling [47]. Most studies measured noise using sound level meters and acoustic audio recorders. Terry et al. (2021) noted taking sound measurements with a smartphone app [46]. Several of the studies also reported noise using A-weighted sound pressure level. No study reported noise findings using other sound frequencies such as C or Z weighting. The majority of studies measured multiple dimensions of noise; however, a minority of studies specifically assessed certain combinations of noise dimensions, cited to be lacking in the field, such as dual investigation of subjective and objective dimensions. Figure 2 displays a bar chart of studies and number of noise dimensions measured.

**Table 2:** Dimensions of noise.

	Qualitative		Quantitative		Subjective				
Article Author (Publication Year)	Noise Level	Noise Events	Noise Type	Noise Source	Noise Sensitivity	Attitudes/Perception of Noise	Noise Audibility	Noise Annoyance	Total Number of Variables Measured
**Bourdeau et al. (2015)** [38]	+ (A-weighted sound level, sound level meter used for fixed-spot and mobile recordings)	−	+	+	−	−	+	−	4
**Buxton et al. (2019)** [50]	+ (Sound exposure level and sound pressure level)	+	+	−	−	−	+	−	4
**Lynch et al. (2011)** [36]	+ (Continuous audio recorder and sound level meter recordings)	+	+	+	−	−	+	−	5
**Betchkal et al. (2023)** [48]	+ (Sound level meter recording)	+	+	+	−	−	+	−	5
**Carter (2014)** [39]	+ (Sound pressure level recordings)	−	+	+	−	−	−	−	3
**Ferguson et al. (2024)** [14]	+ (A-weighted sound pressure level)	−	+	+	+	+	−	+	6
**King et al. (2016)** [40]	+ (A-weighted sound pressure level)	−	+	+	−	−	+	−	4
**King et al. (2018)** [41]	+ (Dosimeters and sound level meter recording A-weighted sound level)	+	−	−	−	−	−	−	2
**Levandowski et al. (2021)** [49]	+ (Acoustic recorders)	−	+	+	−	−	−	−	3
**Pilcher et al. (2009)** [42]	+ (A-weighted sound pressure level)	−	+	+	−	+	+	+	6
**Rice et al. (2022)** [43]	+ (A-weighted sound pressure level)	−	+	+	−	−	−	−	3
**Sheikh and Uhl (2004)** [47]	− (Noise expressed as percent of time heard during sampling, not noise level)	+	+	+	−	−	+	−	4
**Taff et al. (2014)** [44]	+ (Sound level meter, A-weighted summary of aggregate sound level)	−	+	+	−	+	+	−	5
**Terry et al. (2021)** [46]	+ (iPhone app A-weighted sound levels)	−	+	+	−	−	−	−	3
**Yildirim and Ozdil (2016)** [45]	+ (Sound pressure levels)	−	−	−	−	−	−	−	1

Note: “+” indicates that the metric was reported and “−“ indicates it was not reported.

One study measured one dimension of noise. Five studies measured between two to three dimensions of noise. Nine studies measured four or more dimensions of noise. However, it is important to note that only three studies measured at least one objective and one subjective dimension of noise exposure, which has important implications for the applicability of study findings in the context of perception of noise exposure. The lack of majority of the studies, including considerations for the objectivity and subjectivity of noise exposure, is an indicator of a gap for this field of research. Pilcher et al. (2009) assessed noise annoyance (subjective) with multiple objective dimensions, including noise level [42]. Ferguson et al. (2024) was the only study to assess noise sensitivity (subjective) with other objective measures (e.g., noise level) of noise exposure [14]. Taff et al. (2014) assessed the perception and attitude of noise exposure in the park environment and the influence of messaging on the acceptability (subjective) of heard aircraft sound among park visitors, reporting also on the heard noise levels (objective) [44]. These studies were able to directly discuss the acceptability of different noise types among park visitors, which could not be derived solely from objective measurement of noise levels. 

### 3.4. Theme Two: Framing Parks as Venues for Experience: Focus on Park Visitor Perceptions and Insights

The assessment of multiple dimensions of noise exposure is linked to the important objective of understanding the effects of noise exposure on the listener. The three studies that measured a combination of at least one subjective and objective dimension of noise are also the only studies that involved a park visitor survey component in the methodology. Soundscape is an important concept that expands the scope of noise research beyond volume or loudness (i.e., noise levels) and considers the perception of sounds and noises and their meaning [52]. Through this lens, understanding the context of when noise is experienced becomes very important. In essence, in settings such as urban parks, which function as spaces for relaxation, leisure, recreation, and restoration, the perception of noise may be more likely to induce stress and is thus more harmful [53]. The interrelationship between person, place, and activity in space and time impacts perception of the soundscape, and therefore noise pollution in spaces such as urban parks can be deemed as a key agent of diminished environmental quality [14].

Beyond the type of park, examining the association between sources of noise and visitor experience is a key public health query to better understand the impact of the environment on individuals exposed. Ferguson et al. (2024) conducted a study investigating park visitor’s soundscape perception employing subjective and objective measurements at the Muir Woods National Monument [14]. The researchers suggested that park visitors’ subjective experiences were more influential on their perception of the park’s soundscape compared to only acoustic factors like the measured sound level of the park [14]. This finding suggests the importance of a double-pronged approach to studying environmental exposures that are highly subjective in nature such as noise. In the context of noise research, failing to assess the subjective, particularly for outcomes such as noise annoyance, could potentially result in unidimensional conclusions about the full extent of the impact of objective findings.

### 3.5. Theme Three: A Multidisciplinary Scope of Study Implications and Next Steps

In urban parks, understanding the current state of spaces with frequent visitation by city residents requires a multidisciplined effort across multiple sectors including experts in urban planning, landscape architecture, environmental management and safety, and public health. Several of the studies also reported affiliations of the use of data from the National Park Service, which is a nonprofit organization committed to the protection and management of park systems across the US. The discussed implications across all studies in the review differed, with some studies having findings geared toward informing park management interventions and noise control decisions [43,44]. Meanwhile, other studies focused on informing city officials of the value of parks in protecting city residents and pedestrians from dangerous noise pollution levels [40,41]. Two of the studies were specifically targeting regulators of air traffic, by documenting data on the impact of aircraft noise within park spaces [47,48].

### 3.6. Critical Appraisal

To assess the methodological quality of included cross-sectional and observational studies, we applied relevant items from the AXIS Critical Appraisal Tool, which was specifically developed for evaluating cross-sectional research. The AXIS tool comprises twenty items covering key domains such as clarity of study aims, appropriateness of study design, sampling strategy, sample size justification, measurement validity and reliability, response rate reporting, risk of non-response bias, appropriateness of statistical methods, ethical considerations, and declaration of conflicts of interest. Each study was independently appraised for these domains, with particular attention given to clarity of study aims, measurement validity of noise exposure, and reporting of response rates when applicable—all of which are critical elements for assessing the robustness of studies on noise in parks. Discrepancies between reviewers were resolved by consensus. The appraisal revealed that while all studies clearly stated their objectives and employed appropriate cross-sectional designs, when applicable many did not provide sample size justifications or details on non-response bias, limiting confidence in the representativeness of findings. Table 3 is a summary of the appraisal results displaying each item of the tool and whether it was addressed or not applicable for each study. In the review, six studies—without components involving study participants or response rates—were found to have addressed all domains of the AXIS tool appropriately, with a global appraisal rating of strong shown in Table 3.

**Table 3:** AXIS critical appraisal results.

Item	Bourdeau et al. (2015) [38]	Buxton et al. (2019) [50]	Lynch et al. (2011) [36]	Betchkal et al. (2023) [48]	Carter (2014) [39]	Ferguson et al. (2024) [14]	King et al. (2016) [40]	King et al. (2018) [41]	Levandowski et al. (2021) [49]	Pilcher et al. (2009) [42]	Rice et al. (2022) [43]	Sheikh and Uhl (2004) [47]	Taff et al. (2014) [44]	Terry et al. (2021) [46]	Yildirim and Ozdil (2016) [45]
1—Clarity of Aims	✔	✔	✔	✔	✔	✔	✔	✔	✔	✔	✔	✔	✔	✔	✔
2—Appropriate Study Design	✔	✔	✔	✔	✔	✔	✔	✔	✔	✔	✔	✔	✔	✔	✔
3—Sample Justification	✔	✔				✗				✗	✔		✗		
4—Clarity of Target Population						✔				✔	✔		✔		
5—Sample frame from appropriate population						✔				✔	✔		✗		
6—Appropriate sample selection						✔				✗	✔		✗		
7—Measures taken to address non-responders						✗				✗	✔		✗		
8—Outcome variables measured appropriate to aims of the study	✔	✔				✔	✔			✔	✔	✔	✔		
9—Risk factor and/or outcome variables measured correctly	✔	✔				✔	✔			✔	✔	✔	✔		
10—Clarity of what was used to determine statistical significance	✔	✔	✔	✔		✔	✔	✔	✗	✔	✔	✔	✔	✔	✔
11—Methods sufficiently described to enable them to be repeated	✔	✔	✔	✔	✔	✔	✔	✔	✔	✔	✔	✔	✔	✔	✔
12—Basic data adequately described	✔	✔	✔	✔	✔	✔	✔	✔	✔	✔	✔	✔	✔	✔	✔
13—Nonresponse bias concerns addressed						✗				✔	✗		✗		
14—Description of non-responders						✗				✔	✔		✗		
15—Internal consistency of results	✔	✔	✔	✔	✔	✔	✔	✔	✔	✔	✔	✔	✔	✔	✔
16—Results presented for all analyses described in methods	✔	✔	✔	✔	✔	✔	✔	✔	✔	✔	✔	✔	✔	✔	✔
17—Author’s discussions and conclusions justified by results	✔	✔	✔	✔	✔	✔	✔	✔	✔	✔	✔	✔	✔	✔	✔
18—Limitations of study discussed	✔	✔	✗	✔	✔	✔	✔	✔	✗	✔	✔	✗	✔	✗	✗
19—Funding sources or conflicts of interest that may affect authors’ interpretations of results	✗	✗	✗	✗	✗	✗	✗	✗	✗	✗	✗	✗	✗	✗	✗
20—Attained ethical approval or consent						✔				✗	✗		✗		
Global Rating	Strong	Strong	Moderate	Strong	Strong	Weak	Strong	Strong	Moderate	Weak	Moderate	Moderate	Weak	Moderate	Moderate

✔ = yes or addressed, ✗ = no or not addressed, Gray = not applicable; Note that for several studies, due to no study participant component, gray boxes are present.

## 4. Discussion

### 4.1. Contributions to Existing Research

#### 4.1.1. Application of Diverse Measurement Approaches and Technologies

This review included work spanning from the early 2000s to 2024, suggestive of differences in noise measurement approaches over time. Use of smartphones for noise research is not extensively used but could potentially be of interest as technology advances. This is particularly relevant given conventional sound level meters are expensive and require extensive training for accurate utilization [54,55]. In this review, studies varied in approaches to measuring noise, with majority using sound level meters. One study integrated smartphone technology into the noise measurement methodology—Terry et al. (2021) used iPhone measurements with the Lefebvre (2010) SPLnFFT app [46]. The study reported that the iPhone measurements taken with the app were within an accuracy of 2.3 dB compared to reference levels measured by a sound level meter [46]. Two studies conducted in 2016 and 2018 used a fixed spot and mobile approach to measure noise, while for the latter, sound meters were attached to a backpack to capture the exposure and complete acoustic nature of a pedestrian walking in a park. Findings showed an accuracy of the mobile measurements within a three dB (A) difference in fixed-spot measurements [40,41].

The integration of visual assessment of the data was also incorporated across two of the studies, which resulted in the generation of maps displaying the park environments and the coupling of noise level data with the visual data [39,41]. Combining audio data and embedding it into the context of photographs of the park environment allowed more salient representations of the environments under study and implications regarding factors, such as tranquility afforded by the parks, which was a novel contribution [39,41]. Levandowski et al. (2021) utilized a multi-method approach and integrated cameras in addition to acoustic recorders, for the purpose of detecting the state of existing biodiversity in Grand Teton National Park [49]. This study was the only paper that focused on the impacts and role of sound in assessing the ecological patterns within the park [49]. Noise is oftentimes not an isolated exposure and is often co-experienced with other environmental pollutants such as air pollution. King et al. (2016) utilized air pollution monitoring technology in addition to noise level assessment to examine the presence of co-exposure to air and noise pollution among park visitors [40]. These innovative, technology-enhanced approaches underscore the potential for multi-modal data collection to provide richer, more contextually grounded insights into the complex interplay between noise, park environments, and their impacts on both human and ecological health.

#### 4.1.2. Temporal Considerations

Within this review, there was variation in how the effect of time was accounted for in the methodology and study design of the conducted work. From a broad perspective, the notion of how a specific time period in the context of a shift in the status quo of society affects noise in parks was assessed by one study—Terry et al. (2021) investigated how noise levels in parks in Boston differed prior to the COVID-19 pandemic and during the pandemic [46]. Among the three parks investigated, two parks aligned with their hypothesis, with results finding one to three dB lower sound levels during the pandemic. The third park, which differed, had a four to six dB increase in sound levels during the pandemic, and was described as a park transected by a highway, which highlighted the impact of design factors in addition to time period in noise assessment of parks.

Being embedded within temporal considerations of noise exposure is the role of seasonality and the impact of time of year on both noise levels and the user rates of park environments. Research has shown that factors such as temperature, humidity, and wind speed are the main seasonal factors causing the seasonal variations in noise levels [56]. King et al. (2016) and Terry et al. (2021) explicitly created tables in the results or supplementary files of the paper listing factors such as the temperature, humidity, precipitation, and wind speed for each day of noise assessment in their study [40,46]. Among studies that specified the month that the study took place, majority occurred during the fall seasons, which aligns with common seasons for park use, and thus reflect noise exposures that are relevant to the peak use of the park.

Several studies outlined details surrounding conditions of the days when noise measurements were taken, but many differed in what was reported, suggesting a potential need for standardization in reporting protocol for noise measurement testing days. Bourdeau et al. (2015) did specify days of week that noise measurements were taken, Friday through Sunday, and did provide a statement of the meteorological conditions for each testing day in the text [38]. Similarly, Pilcher et al. (2009) and Ferguson et al. (2024) provided exact dates for when phase one measurements of the study survey were conducted but no information regarding the weather was given [14,42]. Yildirim and Ozdil (2016) provided temporal detail in the methods section for noise measurement testing specifying the time of day that measurements were taken (i.e., the sound levels were measured weekdays and weekends 10 am–1 pm and at 4 pm) and provided a broad statement that weather conditions were not severe during measurements [45]. Sheikh and Uhl (2004) measured aircraft noise for 3 hours in each park between 09:00 and 15:00 on weekdays and reported that the temperature varied between 10 °C and 21 °C during the sampling periods [47]. Some studies such as Rice et al. (2022) provided season for when study measures were conducted but do not specificity of time of day or exact days of the week, which used a random sampling approach to determine the days and times for data collection [43].

### 4.2. Limitations of the Evidence Base

#### 4.2.1. Absence of Direct Noise to Stress-Related Health Outcome Measurements

Assessing the link between noise level exposure and psychological perception of noise is a highly complex process. A relevant path to further exploring how noise affects individuals would be direct assessment of health outcomes among the exposed, to capture the auditory and non-auditory health effects. Stress elicitation is cited as a key pathway that noise adversely affects health, with research suggesting that noise annoyance stimulates the stress response [6,57]. Despite the complexity of deriving a causal link between noise and health in environments such as parks which have multiple exposures, evidence on existing associations between noise and health in park users would further strengthen the field. Within all of the studies included in this review, particularly the four studies involving study participants, none directly measured a health outcome suggested by the prior literature to be associated with adverse noise exposure. The broader literature emphasizes the risk of noise exposure in inducing physiological stress [53]. Several of the article’s introductions acknowledge the adverse effects of noise exposure on human health. Across studies, the absence of evidence that enhances understanding of the role of noise in parks on the health of park visitors reveals a current gap in the literature. While the studies evaluating noise in urban parks offer vital and impactful insights into the park environment, the paucity of measured health outcomes among park visitors suggests opportunities for additional studies to further explore. Specifically, measuring how noise in the park environments impacted the perceived stress or physiological stress levels of park users, through collection of biomarkers or physical assessment metrics such as blood pressure, would require additional study logistics, yet it would offer relevant insights.

#### 4.2.2. Generalizability of Sampled Park Visitors

The studies in this review that included a park visitor survey highlighted approaches to sampling park visitors that raised concerns of the limited external validity of the obtained findings. Given that park visitors were often conveniently sampled based on when noise levels were being assessed in the park and as they were exiting the park environments, park users who visit parks during different hours and are representative of additional sociodemographic and economic characteristics may not be reflected in the results of the papers included in this review, for a variety of reasons including time of day, availability at the time of study, and the investigators’ approach to soliciting participants. Additional work outlining tested protocols and recruitment methods for park visitor sampling would be beneficial.

#### 4.2.3. Longitudinal Assessment of Park Environments

A limitation of the articles included in this review is the absence of longitudinal study designs. All studies were cross-sectional in nature, with the studies that reported dates of data collection periods ranging from three days to, at most, two study periods across two years. This lack of longitudinal follow-up restricts the capacity to infer strong conclusions regarding extensive temporal relationships between noise exposure in parks and potential changes in noise levels or visitor experiences with noise over time. The study conducted pre-pandemic and during the pandemic was the only study with repeated measurements that spanned months apart. For the majority of articles in the review, it remains unclear whether observed effects are transient responses to acute noise exposure or reflect more enduring impacts of the park environment, limiting our understanding of how sustained or cumulative noise in park settings may influence visitors’ experiences, health, and overall well-being.

### 4.3. Strengths and Limitations of This Review

#### 4.3.1. Strengths

This scoping review offers several strengths. Primarily, the scoping review addresses a novel research question by thoroughly examining studies focused specifically on noise exposure assessment in urban parks within the United States—a topic that has received limited synthesis despite growing concern about environmental noise in urban settings. Second, by reviewing research spanning over two decades, this review captures the range of research work in methodological approaches, study objectives, and implications, and highlights trends in how noise has been assessed in park environments across various time periods. Also, the inclusion of a critical appraisal of the methodological quality of included studies using the AXIS tool provides important insights into the rigor of existing evidence and identifies key areas where further research would be insightful, such as integration of health outcome measures and longitudinal study designs.

#### 4.3.2. Limitations

Despite its contributions, this review has limitations. Given the specific research aim, the findings and conclusions are only applicable to studies conducted within the United States, limiting generalizability to urban parks in other cultural or geographical contexts. Additionally, inherent to the nature of scoping reviews, this study does not provide systematic bias assessment or meta-analysis, which restricts the ability to estimate pooled effect sizes or strength of associations across studies. Moreover, the search strategy did not include supplementary searches beyond four databases, which may have led to the omission of relevant unpublished reports or dissertations and potentially contributed to publication bias. These limitations should be considered when interpreting the implications of this review for research and policy.

### 4.4. Future Directions and Bridging the Gap Between Physical Sound and Perceived Noise

Parks are vital health promotion and public spaces for communities. Evaluation of noise exposure in park environments is an important park management and public health effort. With growing interests in understanding the full extent of noise exposure impacts on the environment and also human health, it is required to overcome barriers to integrating and measuring perceived noise. The variability of what individuals perceive as noise is shaped by cultural norms, personal history, and characteristics such as preferences, expectations, and socio-demographic attributes, which influence the subjective experiences of humans within park soundscapes [58]. Considering this complex reality underscores the utility of mixed method approaches and interdisciplinary collaboration in merging technical understandings of noise exposure with validation of psychometric tools to link the relationship between objective environmental noise exposure and impacts on park visitors. Ultimately, future work should strive to acknowledge this complexity, while balancing technical precision in capturing noise exposure accurately and understanding the magnitude and breadth of exposure impacts park visitors’ health and experiences in park environments.

## 5. Conclusions

Noise pollution is a significant public health problem, and its intrusion into spaces such as parks poses a major health risk to visitors, warranting careful study. The United States presents a unique context, given its extensive park system and the diverse sources and types of environmental noise. Understanding how noise affects park environments is critical for protecting visitors’ well-being. Evaluating the existing research on noise in parks can help identify knowledge gaps and guide future public health and urban planning efforts.

Ultimately, this scoping review comprehensively maps and synthesizes the existing evidence on noise assessment in urban parks within the United States, addressing a critical gap in understanding how studies have examined the relationships between green space characteristics, measured noise levels, and perceptions of noise among park visitors. By summarizing the current evidence base, highlighting key methodological approaches—including the integration of subjective evaluations of noise exposure—and identifying significant knowledge gaps such as the lack of data on the health effects of park noise and the absence of longitudinal designs, this review provides a foundational understanding of the state of research in this area. These findings emphasize the need for future studies to consider the co-occurrence of noise with other environmental exposures and adopt standardized, multi-modal methodologies to better capture the nuanced impacts of noise in park environments and its influence on the health of park visitors. Collectively, these insights can inform the development of evidence-based policies and interventions aimed at enhancing the acoustic quality and restorative potential of urban and national parks.

## Data Availability

No new data were created or analyzed in this study. Data sharing is not applicable to this article.

## Supplementary Materials

The following supporting information can be downloaded at https://www.mdpi.com/article/10.3390/ijerph22121882/s1. Supplementary Material S1. Search Strategy. Supplementary Material S2. Screening Checklist for Records.
