# Supplementary material for: Assessment of Noise Exposure in United States Urban Public Parks: A Scoping Review
Source: Int J Environ Res Public Health. 2025 Dec 18;22(12):1882. doi: 10.3390/ijerph22121882 (PMC12733263; doi:10.3390/ijerph22121882)
Supplement: Supplementary file 1 [file ijerph-22-01882-s001.zip › ijerph-3894161-supplementary.pdf]

SUPPLEMENTARY MATERIALS

Supplementary Material S1. Search Strategy

Table 1. Search strategy for the database search conducted on October 1, 2024.

ID	Search	Hits
1	**Parks:** “Parks, Recreational”[Mesh] OR “Recreational Park” [tiab] OR “Recreational Parks” [tiab] OR “Urban Parks” [tiab] OR “Urban Park” [tiab] OR “Community Parks” [tiab] OR “Community Park” [tiab] OR “Green Space*” [tiab] OR “greenspace*” [tiab] OR “parks” [tiab] OR “National Park” [tiab]<br><br>**Recreation/ Leisure:** “leisure activities”[MeSH Terms] OR “leisure activities”[tiab] OR “recreation”[MeSH Terms] OR “recreation”[tiab] OR “recreational” [tiab]	21,085
2	**Urban:** “cities”[MeSH Terms] OR “cities”[tiab] OR “city”[tiab] OR “urban” [tiab]	489,766
3	**Noise:** “noise” [mesh] OR “noise, transportation” [mesh] OR “noise” [tiab]<br><br>**Sound:** “Sound”[Mesh:NoExp] OR “sound pollution” [tiab] OR “soundscape” [tiab] OR “soundscapes”[tiab]	204,433
4	#1 AND #2	21,085
5	#3 AND #4	2,423

Supplementary Material S2. Screening Checklist for Records

Place Criteria

Was the study conducted in a public park?

Y **N**

(IF NO, EXCLUDE)

Intervention/Exposure

Did the study assess noise exposure/noise pollution objectively?

Y **N**

(IF NO, EXCLUDE)

Control/Comparison

Not applicable

Y **N**

(IF NO, EXCLUDE)

Outcome(s)

Did the study report measured noise?

Y **N**

(IF NO, EXCLUDE)

Study Design

Did the study use a quantitative evaluation study design?

Y **N**

(IF NO, EXCLUDE)
